# Oxidized plasma albumin promotes platelet-endothelial crosstalk and endothelial tissue factor expression

**DOI:** 10.1038/srep22104

**Published:** 2016-02-24

**Authors:** Lisa Pasterk, Sandra Lemesch, Bettina Leber, Markus Trieb, Sanja Curcic, Vanessa Stadlbauer, Rufina Schuligoi, Rudolf Schicho, Akos Heinemann, Gunther Marsche

**Affiliations:** 1 Institute of Experimental and Clinical Pharmacology, Medical University of Graz, Austria; 2 Division of Gastroenterology and Hepatology, Department of Internal Medicine, Medical University of Graz, Austria.

## Abstract

Plasma advanced oxidation protein products (AOPPs), a class of pro-inflammatory pathogenic mediators, accumulate in subjects with chronic kidney disease. Whether AOPPs contribute to coagulation abnormalities, which are frequently seen in uremic patients, is unknown. Here we report that AOPPs activate platelets via a CD36-mediated signaling pathway. Activation of signaling pathways by AOPP-platelet interaction resulted in the expression of several platelet activation markers and rapidly induced the expression of CD40 ligand, triggering platelet adhesion to endothelial cells and promoting endothelial tissue factor expression. AOPPs and serum tissue factor levels were considerably increased in end stage renal disease patients on hemodialysis and a significant correlation of AOPPs and serum tissue factor was found. Interestingly, serum levels of AOPPs and tissue factor were substantially lower in stable kidney transplant patients when compared with hemodialysis patients. Given that CD36 is known to transduce the effects of oxidized lipids into platelet hyperactivity, our findings reveal previously unknown pro-thrombotic activities of oxidized plasma albumin via a CD36 dependent pathway.

Renal insufficiency is independently associated with elevations in inflammatory and procoagulant biomarkers, and patients with chronic kidney disease experience substantial increased mortality and morbidity as a consequence of thromboembolic events^1^. Oxidative stress has emerged as a constant feature of chronic renal failure evidenced by an overabundance of lipid, carbohydrate, and advanced oxidation protein products (AOPPs) in the plasma and tissues of uremic patients^2^. Reactive chlorinating species generated by myeloperoxidase of activated neutrophils are thought to be a major pathway for the formation of AOPPs^3^,^4^. AOPPs are thought to be potent mediators of vascular inflammation and were shown to interfere with high-density lipoprotein metabolism^3^,^5^,^6^,^7^,^8^,^9^,^10^. Previous studies have shown that plasma albumin may consume the majority of chlorinated oxidants with limited damage to other materials^11^. Reactive chlorinating species modify proteins in various ways, including conversion of cysteine residues to disulphides and higher oxidation products, conversion of methionine residues to methionine sulphoxides, oxidation of tryptophan and chlorination of amino groups and tyrosine^9^,^12^,^13^. Massive oxidation of plasma albumin was demonstrated by mass spectrometry in primary nephritic syndrome, involving almost complete sulphonation of the free sulfhydryl group Cys34 of albumin^14^,^15^. A growing body of evidence suggests that AOPPs could be considered as a new class of renal pathogenic mediators^3^,^16^,^17^,^18^. As chronic kidney disease is associated with oxidative stress and AOPPs formation^19^,^20^,^21^,^22^,^23^, we assessed in the present study whether the interaction of AOPPs with platelet scavenger receptors alter platelet reactivity, inducing pro-thrombotic signals.

## Results

### AOPPs activate platelets

*In vivo*, the generation of chlorinated oxidants is a feature of phagocytic cells that possess myeloperoxidase, the only enzyme that is able to generate a chlorinated oxidant. *In vitro*, AOPPs can be formed by exposure of albumin to the neutrophil product hypochlorous acid^3^,^6^,^10^. First, we tested whether oxidative modifications transform plasma albumin into platelet activators. For this purpose, platelets isolated from plasma of healthy donors were exposed to *in vitro* generated AOPPs, an *in vivo*–relevant model of modified albumin found in patients suffering from chronic kidney disease^3^. The AOPP content of 1 mg/mL of *in vitro*-generated AOPP-albumin was 7.0 μmol/L and was comparable to the AOPP content of isolated albumin from end stage renal disease patients which ranges from 2 to 10 μmol/L^10^.

**Figure 1:**
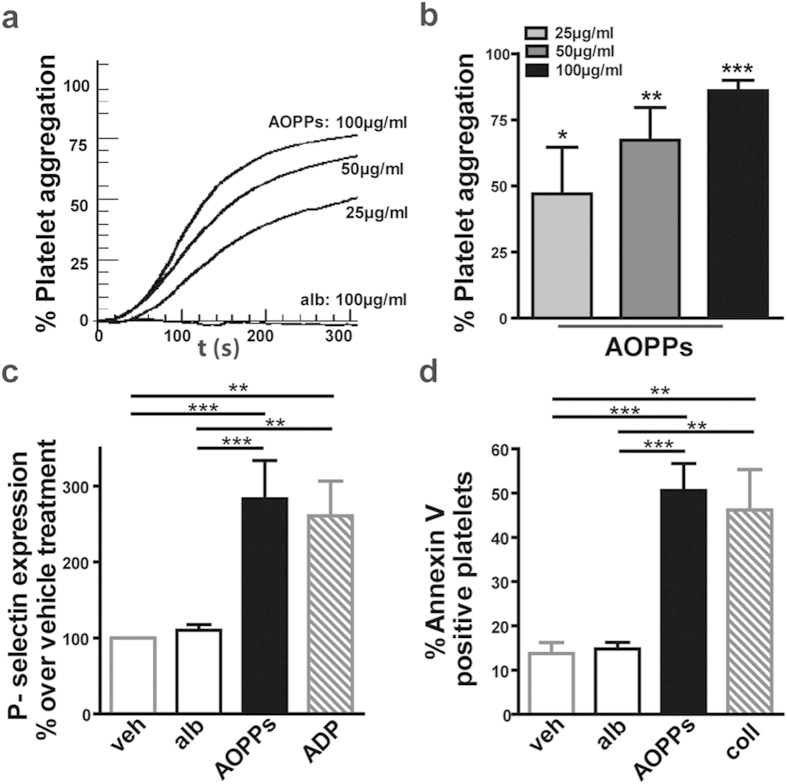
AOPPs induce platelet aggregation and upregulate P-selectin and phosphatidylserine surface expression on platelets. (**a,b**) Platelets were preincubated with albumin (alb) or AOPPs at indicated concentrations. A representative recording is shown in (**a**). Mean values of 4 independent experiments are expressed as percent of maximal platelet aggregation in (**b**). (**c**) Upregulation of P-selectin was assessed in platelets by flow cytometry. Platelets were treated with albumin (100 μg/mL), AOPPs (100 μg/mL) or ADP (3 μM) in the presence of cytochalasin B (5 μg/mL) and normalized to vehicle treated platelets (n = 7). (**d**) Platelets were exposed to vehicle (veh), albumin (100 μg/mL), AOPPs (100 μg/mL) or collagen (coll, 10 μg/mL) and the percentage Annexin V-positive platelets, was determined by flow cytometry (n = 7). All values are shown as mean + SEM. *P < 0.05, **P < 0.01, ***P < 0.001 as indicated.

Strikingly, AOPPs (25 – 100 μg/mL) potently triggered platelet aggregation in a concentration-dependent manner (Fig. 1a,b). To ensure that AOPPs induce an activation response, experiments were performed in the presence of EGTA. Addition of EGTA (1 and 5 mmol/L) dose dependently inhibited AOPPs induced platelet aggregation, excluding AOPPs induced platelet agglutination (Supplementary Figure). AOPPs (100 μg/mL) promoted the expression of the platelet activation marker P-selectin comparable to stimulation of platelets with ADP (3 μM) in the presence of cytochalasin B (Fig. 1c). The conversion of platelets from an activated to a procoagulant state is associated with specific morphological changes including phosphatidylserine exposure on the surface of activated platelets. AOPPs (100 μg/mL) significantly increased the percentage of platelets that were stained positive with Annexin V comparable to collagen (10 μg/mL) stimulated platelets, indicating increased phosphatidylserine expression on the surface (Fig. 1d).

### Platelet scavenger receptor CD36 promotes AOPPs induced effects

**Figure 2:**
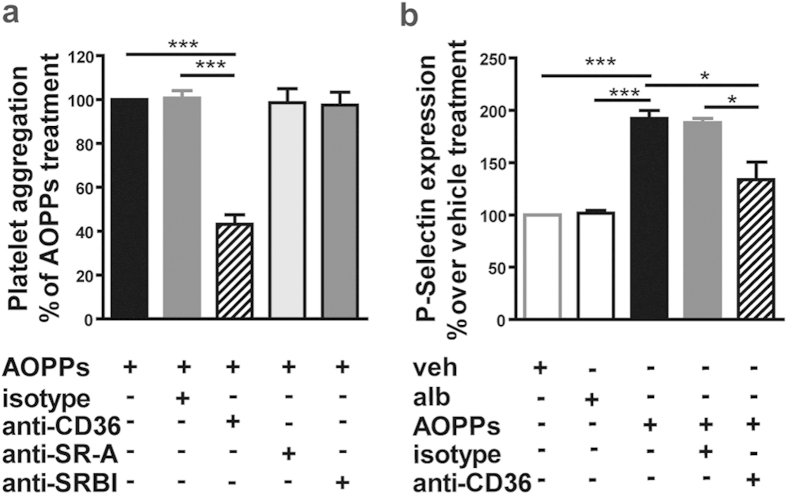
CD36 is involved in AOPPs induced platelet aggregation and P-selectin expression. (**a**) Platelets were pretreated with an anti-CD36 blocking antibody (4 μg/mL), an anti-SR-A blocking antibody (4 μg/mL), an anti-SRBI blocking antibody (4 μg/mL) and a respective isotype control before addition of AOPPs (100 μg/mL) to platelets. Data are expressed as percent of normalized platelet aggregation (n = 3–7). (**b**) P-selectin surface expression in the presence of an anti-CD36 blocking antibody (4 μg/mL) or respective isotype control was measured in albumin (alb) or AOPPs (100μg/mL) treated platelets and normalized to vehicle treated platelets (n = 3–6). All values are shown as mean + SEM. *P < 0.05 and ***P < 0.001 as indicated.

Next we assessed whether scavenger receptors mediate AOPPs induced effects. AOPPs are known ligands of class B scavenger receptors SR-BI and CD36^8^,^9^,^10^, and both receptors are expressed on platelets^24^,^25^. Of note, a blocking antibody against CD36 that specifically blocks an epitope on CD36 responsible for the oxidized LDL-binding domain of CD36, markedly inhibited AOPPs induced platelet aggregation, whereas blocking antibodies against SR-BI and SR-A showed no effect (Fig. 2a). Blockade of CD36 also effectively inhibited P-selectin expression in response to AOPPs (Fig. 2b), providing evidence that platelet CD36 is involved in AOPPs induced platelet activation.

### AOPPs trigger reactive oxygen species (ROS) production in platelets

**Figure 3:**
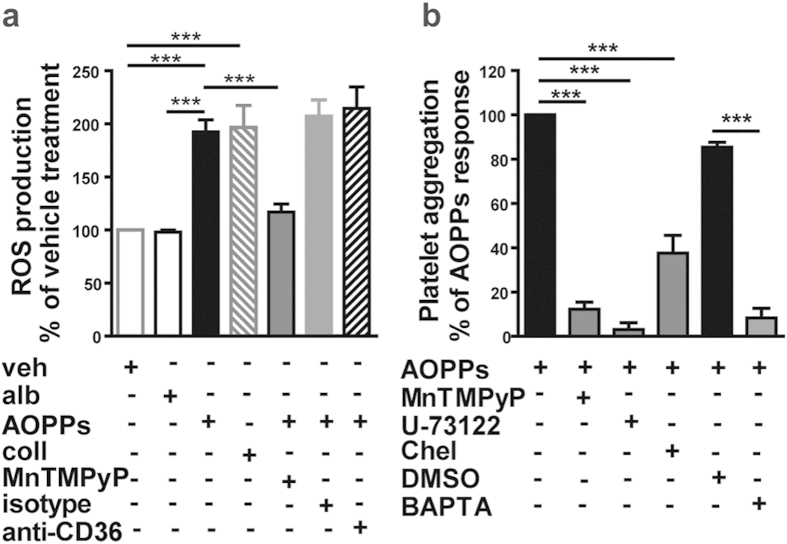
AOPPs induced platelet aggregation depends on ROS production and PLC and PKC activation. (**a**) ROS production of platelets treated with vehicle (veh), collagen (coll, 10 µg/mL) albumin (alb, 100 μg/mL) or AOPPs (100 μg/mL), with or without pretreatment with the superoxide dismutase mimetic MnTMPyP (50 μmol/L), anti-CD36 blocking antibody or the respective isotype control. ROS production was assessed by flow cytometry and is depicted as % of vehicle treatment (n = 3). (**b**) MnTMPyP (50 μmol/L), the PLC inhibitor U-73122 (3 μmol/L), the PKC inhibitor chelerythrine (Chel; 10μmol/L), the Ca^2+^ scavenger BAPTA-AM (BAPTA; 20 μmol/L) and dimethyl sulfoxide (DMSO) were added to platelets prior to stimulation with AOPPs (100 μg/mL). Inhibition of platelet aggregation is shown as percent of normalized AOPPs response. BAPTA-AM was dissolved in DMSO, therefore effects of BAPTA-AM were compared to AOPPs induced response in the presence of DMSO (n = 3–5). All values are shown as mean + SEM. ***P < 0.001 as indicated.

Activated platelets generate ROS, which promote a pro-thrombotic state^26^. Notably, exposure of platelets to AOPPs (100 μg/mL) increased ROS/superoxide production to a similar extend when compared with collagen (10 μg/ml), whereas control albumin showed no effect (Fig. 3a). Unexpectedly, blocking of CD36 did not alter intracellular ROS levels (Fig. 3a) suggesting a role of additional receptors in AOPPs induced ROS production. Nevertheless, when platelets were pretreated with the superoxide dismutase mimetic MnTMPyP, AOPPs induced ROS production as well as platelet aggregation was almost completely abolished (Fig. 3b), suggesting that ROS production plays a role in AOPPs induced platelet activation.

Previous studies have shown that phospholipase C (PLC) as well as protein kinase C (PKC) activation are linked to intracellular ROS production in different cell types^8^,^27^,^28^. Classical PKCs play a critical and general role in platelet granule secretion and subsequent aggregation^29^. We observed that AOPPs induced platelet aggregation was inhibited by the PLC inhibitor U-73122, the PKC inhibitor chelerythrine and the Ca^2+^ scavenger BAPTA-AM (Fig. 3b).

### AOPPs trigger platelet CD40 ligand (CD40L) expression, induce platelet adherence to HCAECs under flow and promote endothelial tissue factor (TF) expression

**Figure 4:**
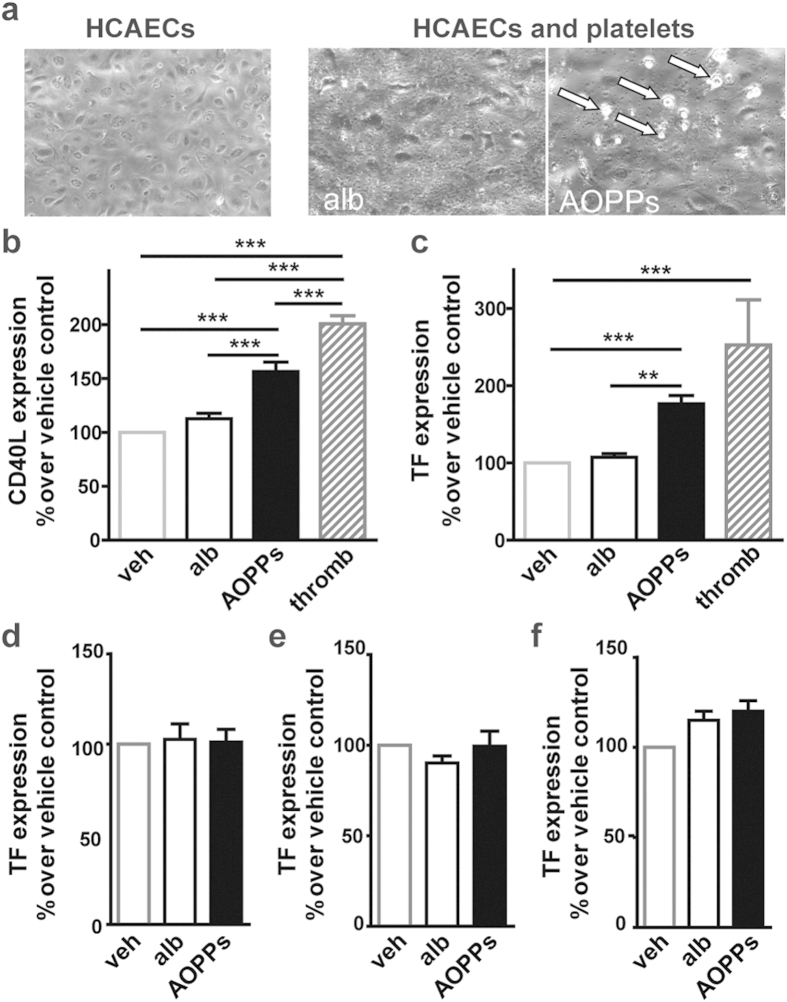
AOPPs induce CD40L expression on platelets, increase adhesion to HCAECs and promote endothelial tissue factor expression on HCAECs. (**a**) Platelets were treated with albumin (alb, 100μg/mL) or AOPPs (100 μg/mL) and perfused over a monolayer of human coronary aortic endothelial cell (HCAEC, left panel) with constant shear stress. Adhesion of platelets to HCAECs is shown in the right panel. Platelet aggregates are indicated by arrows. Images were taken 6 minutes after the start of perfusion (n = 3) (**b**) Platelets were preincubated with vehicle (veh), albumin (100 μg/mL), thrombin (thromb, 200 U/mL) or AOPPs (100 μg/mL) and CD40L expression was assessed by flow cytometry. Data indicate the percentage of CD40L expression over vehicle treatment (n = 5). (**c**) Platelets were exposed to vehicle (veh), albumin (100μg/mL) or AOPPs (100 μg/mL) and incubated with HCAECs (n = 3). (**d**) Supernatants of platelets that were exposed to vehicle, albumin (100μg/mL) or AOPPs (100 μg/mL) were incubated with HCAECs. (n = 3–4) (**e**) HCAECs were incubated with vehicle, albumin (100 μg/mL) or AOPPs (100 μg/mL) in the absence of platelets (n = 3–4). (**f**) Washed platelets were incubated with albumin (100 μg/mL) or AOPPs (100 μg/mL). (**c–f**) TF expression on HCAECs or platelets (**f**) was measured by flow cytometry and depicted as percentage TF expression over vehicle treatment. All values are shown as mean + SEM. ***P < 0.01 as indicated.

Interactions between platelets and endothelial cells are involved in all stages of atherosclerotic disease^30^. Platelets express the ligand of CD40 (CD154) within seconds of exposure to agonists^31^, and interact with endothelial cells to participate directly in the induction of an inflammatory response. Notably, AOPPs induced platelet CD40L (Fig. 4b) expression and promoted increased platelet adherence to human coronary aortic endothelial cells (HCAECs) by forming aggregates on HCAECs as indicated in Fig. 4a, associated with a considerable induction of tissue factor expression of HCAECs (Fig. 4c). Thrombin (200 U/mL) was used as a positive control (Fig. 4b,c). When HCAECs were exposed to supernatants of AOPPs stimulated platelets, no endothelial tissue factor expression was observed (Fig. 4d) and AOPPs did not induce endothelial tissue factor expression in the absence of platelets (Fig. 4e). Moreover, platelets did not express tissue factor upon AOPPs treatment (Fig. 4f), strongly suggesting that direct platelet-endothelial interaction is needed to trigger tissue factor expression.

### Serum levels of tissue factor and AOPPs are linked in uremic patients

**Figure 5:**
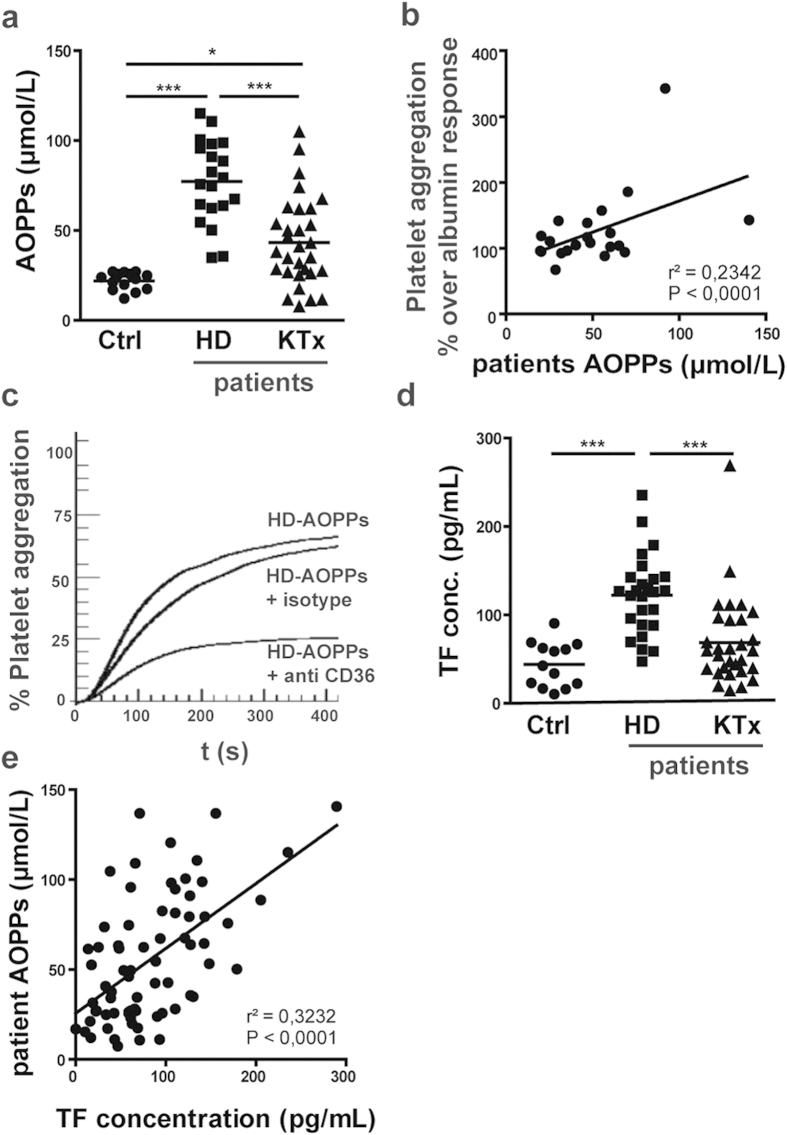
AOPPs isolated from end stage renal disease patients are proaggregatory: Serum AOPP levels correlate with TF concentration in uremic patients. (**a**) AOPP levels of serum samples from end stage renal disease patients on hemodialysis (n = 28, HD), subjects after kidney transplantation (n = 29, KT×) and control subjects (n = 14, Ctrl). (**b**) Platelets were incubated AOPPs (1mg/mL) isolated from end stage renal disease patients on hemodialysis (n = 21) and aggregation was induced with ADP (5–20 μM) in the presence of fibrinogen (5 μg/mL). ADP concentration was chosen to yield 30–50% aggregation in the presence of control albumin (1 mg/mL). Correlation between serum levels of AOPPs and platelet aggregation over control albumin is shown. (**c**) An anti-CD36 antibody (4 μg/mL) or respective isotype control was added to platelets prior to addition of AOPPs (1mg/mL) isolated from an end stage renal disease patient on hemodialysis. (**d**) Tissue factor (TF) concentration measured in plasma from end stage renal disease patients on hemodialysis (n = 28), subjects after kidney transplantation (n = 29) and controls (n = 14). (**e**) Correlation between serum levels of AOPPs and tissue factor concentration. Statistical significance is given as *P < 0.05 and ***P < 0.001.

The clinical characteristics of study subjects are given in Table 1. Serum AOPP levels were markedly higher in end stage renal disease patients on hemodialysis (HD) when compared with healthy controls (Fig. 5a). Interestingly, AOPP content in kidney transplant recipients (KTx) was significantly lower when compared to end stage renal disease patients on HD, indicative of substantially lower oxidative stress (Fig. 5a). In good agreement with results above, AOPP-albumin isolated from uremic patients promoted ADP induced platelet aggregation dependent on AOPP levels (Fig. 5b). Patients AOPPs induced platelet aggregation was inhibited by blocking CD36 (Fig. 5c). Tissue factor levels were profoundly increased in end stage renal disease patients on HD whereas kidney transplant patients showed levels that were comparable to controls (Fig. 5d). Of particular interest, serum tissue factor and serum AOPP levels correlated significantly in uremic subjects (Fig. 5e).

## Discussion

**Figure 6:**
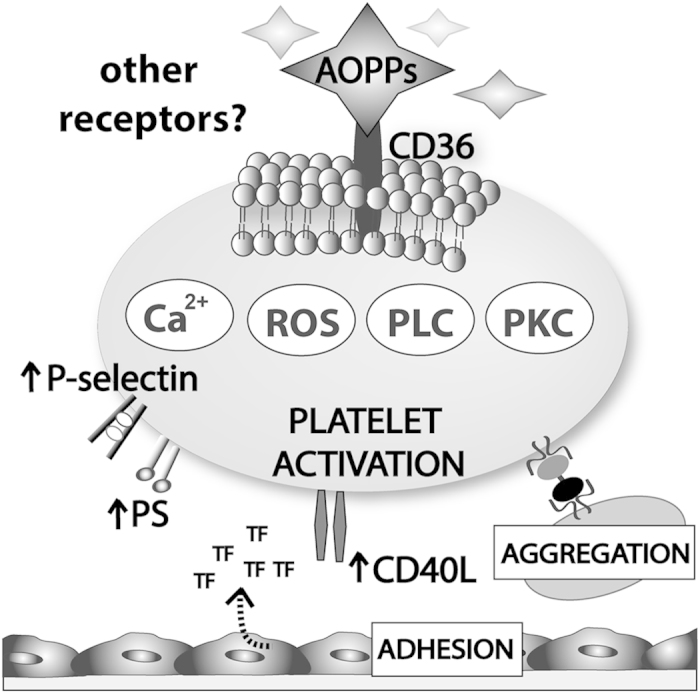
Proposed prothrombotic activities of AOPPs. AOPPs promote platelet activation via platelet scavenger receptor CD36, induce intracellular signaling involving ROS production, activation of PLC and PKC resulting in calcium mobilization. AOPPs promote the expression of the platelet activation markers P-selectin, phosphatidylserine (PS), CD40L and platelet adhesion to fibrinogen and endothelial cells. Tissue factor (TF) expression is induced by direct platelet-endothelial interaction.

In the present study, we provide several lines of evidence that AOPPs show previously unknown pro-thrombotic activities via a platelet CD36-mediated, redox-dependent pathway (Fig. 6).

CD36 was recognized as a major platelet glycoprotein more than 3 decades ago, but its role in platelet physiology had remained obscure for a long time. CD36 recognizes a number of distinct ligands including thrombospondin-1, oxidized phospholipids present in oxidized low-density lipoproteins, fatty acids, microbial diacylglycerides and many others^32^. We provide evidence that AOPP-albumin isolated from end stage renal disease patients as well as *in vitro* generated AOPPs markedly increase ADP-induced platelet aggregation via CD36.

Our results suggest that AOPPs (like most platelet agonists) activate PLC, which catalyzes the hydrolysis of phosphatidyl inositol 4, 5 bisphosphate to inositol triphosphate (IP3) and diacylglycerol. Diacylglycerol further activates Ca^2+^ mobilization and PKC, respectively. Classical PKCs, particularly PKC a, play a critical and general role in platelet granule secretion and subsequent aggregation^29^. Previous studies demonstrated that AOPPs activate NAD(P)H oxidase through a PKC dependent pathway and promote ROS production in various cell types^8^,^28^,^33^. It remains to be determined whether PKC is linked to ROS production in platelets. AOPPs induced platelet aggregation was abolished by the superoxide dismutase mimetic MnTMPyP, suggesting a role of intracellular ROS formation in platelet activation. Unexpectedly, AOPPs triggered ROS production in platelets was not altered in the presence of a CD36 receptor blocking antibody, suggesting that additional receptors are involved AOPPs induced ROS formation. AOPPs increased platelet P-selectin expression, a critical mediator of platelet-leukocyte interaction that promotes neutrophil transendothelial migration^34^,^35^. Moreover, AOPPs increased the surface expression of the late platelet activation marker phosphatidylserine. The resulting negatively charged surface of activated platelets is thought to promote the assembly of the prothrombinase complex that accelerates thrombin generation^36^,^37^. Interestingly in this regard, increased surface expression of phosphatidylserine was observed in platelets of chronic kidney disease patients^38^. Furthermore, AOPP-albumin induced the expression of CD40 ligand, likely contributing to the increased platelet adhesion to endothelial cells and thereby stimulating endothelial tissue factor expression, a central player in the initiation of blood coagulation^39^. Importantly, elevated levels of soluble CD40 ligand were shown to increase the risk of cardiovascular events in patients with coronary artery disease^40^. Tissue factor binds factor VIIa resulting in the activation of factor IX and factor X, ultimately leading to fibrin formation. Up-regulation of tissue factor might therefore drive a thrombosis-inflammation circuit promoting cardiovascular complications^41^. Of particular interest, impaired endogenous thrombolysis was recently identified to be strongly associated with cardiovascular events in end stage renal disease patients^42^ and an association between mortality and reduced clot permeability was reported^43^. Remarkably, we observed that a direct platelet-endothelial interaction is needed to induce tissue factor expression, since direct activation of human coronary endothelial cells with AOPP or addition of supernatants of activated platelets failed to stimulate endothelial tissue factor expression.

In good agreement with our *in vitro* data, AOPPs and serum tissue factor levels were markedly increased in end stage renal disease patients on hemodialysis and correlated significantly with each other. A major finding of our study was that serum levels of AOPPs and tissue factor in stable transplant patients were significantly lower when compared with patients on hemodialysis. Kidney transplantation is the preferred therapy for the majority of patients with end stage renal disease because overall survival and quality of life are better than with hemodialysis^44^,^45^; hence reduced serum levels of AOPP-albumin as well as tissue factor might be one of the factors contributing to the decreased cardiovascular mortality of kidney transplant patients.

In conclusion, our present findings reveal previously unknown pro-thrombotic activities of oxidized plasma albumin. Given that AOPPs are recognized by several scavenger receptors involved in cardiovascular disease, including CD36, the receptor for advanced glycation end-products and SR-BI^9^,^10^,^33^,^46^, AOPPs might play a considerable role in the onset and progression of cardio-thrombotic diseases.

## Material and Methods

ADP, collagen and thrombin were purchased from Probe&Go (Osburg, Germany). Bovine serum albumin (BSA, Fraction V) was purchased from GE Healthcare (Buckinghamshire, UK). The phospholipase C (PLC) inhibitor U-73122, protein kinase C (PKC) inhibitor chelerythrine, BAPTA-AM, fibrinogen, fibronectin, EGTA and cytochalasin B were purchased from Sigma (Vienna, Austria). Monoclonal anti-CD36 antibody [clone FA6-152] (ab17044), polyclonal anti-SR-A antibody (ab123946) and anti-IgG1 antibody were purchased from Abcam (Cambridge, UK), polyclonal anti-SRBI antibody (NB400-101) from Novus biological (Cambridge, UK).

The superoxide dismutase mimetic MnTMPyP pentachloride was from Santa Cruz (California, USA). Assay buffer used in P-selectin expression staining was Dulbecco’s modified phosphate-buffered saline (PBS; with or without 0.9 mM Ca^2+^ and 0.5 mM Mg^2+^; Invitrogen, Vienna, Austria). The antibodies against P-selectin, CD40L, TF and IgG1 were purchased from Becton Dickenson (Vienna, Austria). Fixative solution was prepared by mixing 30 ml FACS-Flow with 9 ml aqua dest. and 1 ml CellFix.

### Blood collection

The study was approved by the Institutional Review Board (Ethics committee of the Medical University Graz). Blood was taken from end stage renal disease patients on haemodialysis prior dialysis sessions, from stable kidney transplant patients and from age- and sex-matched control subjects after they signed an informed consent form in agreement with the Institutional Review Board of the Medical University of Graz. All methods were carried out in accordance with the approved guidelines.

### Platelet preparation

For washed platelet preparation, whole blood from healthy donors was collected using sodium citrate (3.8%) as anticoagulant and platelet rich plasma was prepared by centrifugation at 400 × g for 20 min as described^47^,^48^. Subsequently, platelets were rinsed twice with a low pH buffer (140 mM NaCl, 10 mM NaHCO_3_, 2.5 mM KCl, 0.9 mM Na_2_HPO_4_, 2.1 mM MgCl_2_, 22 mM C_6_H_5_Na_3_O_7_, 0.055 mM glucose and 0.35% BSA, pH = 6.5) and centrifuged for 15 min at 1000 × g. The pellet was resuspended in tyrode buffer (10 mM HEPES, 134 mM NaCl, 1 mM CaCl_2_, 12 mM NaHCO_3_, 2.9 mM KCl, 0.34 mM Na_2_HPO_4_, 1 mM MgCl_2_ and 0.055 mM glucose, pH = 7.4). These washed platelets were used for functional platelet assays.

### Isolation of albumin from serum

Blood was taken from end stage renal disease patients on hemodialysis prior dialysis sessions, from stable kidney transplant patients and from age- and sex-matched control subjects as described^9^ in agreement with the Institutional Review Board of the Medical University of Graz. Albumin from HD-patients and controls was separated from other plasma proteins by affinity chromatography using HiTrap Blue HP, 1 mL columns (GE Healthcare) according to the instructions of the manufacturer.

### AOPPs assay

The AOPPs - assay was performed as previously described^49^. In brief, serum was depleted of apoB-containing lipoproteins with polyethylenglycol (PEG). 400 μL PEG-solution (20% PEG in 200 mmol/L glycine, pH = 7.4) was added to 1 mL serum and incubated for 20 min. Precipitate was pelleted by centrifugation (10.000 × g, 30 min) and the supernatant used for AOPP detection. Subsequently, 10 μL apoB-depleted serum was mixed with 40 μL 0.2 mol/L citrate buffer and incubated for 2 min on a shaker. Afterwards, absorbance at 340 nm was measured on a Nano Drop 1000 (Peqlab, Erlangen, Germany) spectrophotometer. Absorbance was converted into the respective AOPP concentrations by means of a standard curve ranging from 1 to 100 μmol/L chloramine-T as described^3^,^9^,^10^. AOPP concentrations were expressed as μmol/L of chloramine-T equivalents.

### *In vitro* AOPPs preparation

AOPPs were prepared by incubation of albumin with hypochlorous acid in the absence of free amino acid/carbohydrates/lipids to exclude the formation of advanced glycation end products (AGEs)-like structures as previously described^9^,^10^. The modified albumin preparations were passed over PD MiniTrap G-25 columns to remove excess reactants and immediately stored at −70 °C until further use.

### Platelet aggregation

Platelet aggregation was recorded at 37 °C with constant stirring using the 4-channel platelet aggregometer APACT4004 (LABiTec, Ahrensburg, Germany) as previously described^48^,^50^. Platelet aggregation of washed platelets was induced with AOPPs (25–100 μg/mL). In experiments using isolated albumin of end stage renal disease patients (patients AOPPs), platelets were pre-incubated for 10 min with patient AOPPs, subsequently ADP (5–20 μM) was added in the presence of fibrinogen (1 μg/mL). The concentration of ADP was adapted to yield 30–50% platelet aggregation in the presence of pooled albumin preparations that were isolated from healthy subjects. Platelet aggregation was measured for 4 min. The blocking antibodies/inhibitors were added 10 min before *in vitro* prepared AOPPs or patient AOPPs was added. Data were expressed as percent of maximum light transmission, with non-stimulated washed platelets being 0% and tyrode buffer 100% or normalized to (patient) AOPPs.

### P-selectin and CD40L surface expression

The experiments were carried out according to previous protocols^48^,^50^. In brief, washed platelets were incubated with AOPP-albumin (100 μg/mL) for 5 min. To compare levels of activation, P-selectin expression was induced with ADP (3 μM) in the presence of cytochalasin B (5 μg/ml) and CD40L was stimulated with thrombin (200 U/mL). Inhibitors/blocking antibodies were added 20 min before platelet stimulation. For P-selectin and CD40L staining, platelets were incubated for 30 min at room temperature in the presence of an anti-CD62P-FITC (15 μg/mL) or anti-CD40L-FITC (15 μg/mL). The samples were washed and fixed, and P-selectin and CD40L expression was detected by flow cytometry.

### Phosphatidylserine surface exposure

To measure phosphatidylserine exposure to the surface of platelets, washed platelets (3 × 10^7^/mL) were incubated for 10 min with albumin, AOPPs (100 μg/mL) or collagen (10 μg/ml), respectively. To each sample 2.5 μl FITC labelled Annexin V was added and platelets were incubated for further 15 min at room temperature in the dark. Platelets were washed and re-suspended in binding buffer according to the manufacturer’s protocol (BD Biosciences, Germany) and phosphatidylserine exposure on platelets was measured as Annexin V staining within one hour using flow cytometry as described^51^.

### Intracellular ROS production

Washed platelets (1 × 10^8^/mL) were mixed with superoxide detection probe (Enzo Life Sciences, Lausen, Switzerland) and incubated according to the manufacturer’s protocol. Subsequently, platelets were pre-incubated with vehicle, MnTMPyP (50 μg/mL) or blocking antibodies, respectively. This was followed by incubation with AOPPs (100 μg/mL) or collagen (10 μg/mL) for additional 15 minutes at room temperature. ROS production was assessed as an increase in fluorescence intensity (FL-2 channel) by flow cytometry.

### Platelet adhesion under flow

For platelet adhesion experiments to human coronary artery endothelial cells (HCAECs) under flow conditions, Vena 8 Endothelial + biochips (Cellix Ltd, Dublin, Ireland) were coated with fibronectin (20 μg/mL) at 4 °C overnight. On the next day, the chip was first rinsed with Dulbecco’s modified PBS and HCAECs (75.000 cells in 14 μL medium) were seeded in each channel. Cells were incubated for 20 min at 37 °C to allow cells to attach to the channels; subsequently medium was added for 2 hours at 37 °C. Washed platelets were incubated with albumin or AOPPs (100 μg/mL) and immediately perfused over the HCAEC monolayer at constant shear stress of 0.5 dyne cm^2^ for 6 minutes using the Mirus nanopump (Cellix). Platelet adhesion was recorded on an Olympus IX70 fluorescence microscope and an Olympus UPlanFI-20/0.40 lens, using a Hamamatsu ORCA-ER digital camera and the Olympus CellP software. Cell images of 3 microscopic fields from each channel were captured.

### Cell culture of human coronary artery endothelial cells (HCAECs)

HCAECs were cultured as previously described^52^,^53^. Cells were purchased from Lonza (Verviers, Belgium) and cultured in EGM-2 MV Bullet medium (Lonza) containing 5% fetal bovine serum at 37 °C in humidified 5% CO_2_. Endothelial cells from two different HCAEC donors were passaged at 80–90% confluence and were used from passage 6–8. 50.000 cells were plated on 48-well plates (Greiner, Germany) and used for experimental procedures after reaching confluence.

### Determination of tissue factor

Washed platelet suspensions were treated with albumin, AOPPs (100 μg/mL) or thrombin (200 U/mL) for 10 min, subsequently diluted 1:1 with endothelial growth media, added to confluent HCAECs and incubated for 3 hours at 37 °C. For control experiments, albumin or AOPPs was directly added to HCAECs in the absence of platelets or the supernatant of albumin or AOPPs treated platelets was co-cultivated with HCAECs. Prior to incubation, culture plates were centrifuged for 2 min at 750 × g to allow cell-cell inter-action. After incubation, supernatants were removed and detachment buffer was added (Dulbecco’s modified PBS with 25 mM HEPES and 10 mM EDTA) for 30 min at 37 °C. The well content was collected and after a washing step (5 min, 400 × g) cell pellets were incubated with anti-tissue factor antibody (0,5 μg/mL) for 30 min at 4 °C. Following a further washing step, cells were resuspended in fixative solution and measured by flow cytometry. For experiments where TF expression on platelets was determined, AOPPs (100 μg/mL) was added to freshly prepared washed platelets and platelets were incubated for 30 min in the presence of an anti-tissue factor antibody (0.5 μg/mL). Further steps were performed as described for P-selectin expression staining. Serum tissue factor levels of uremic patients and controls were determined using the IMUBIND ELISA kit (American Diagnostica Inc, Stamford, CT) according to the manufacturer’s protocol.

### Statistical analysis

Data are shown as mean + SEM for n observations using platelets from different donors. Comparisons of groups were performed using one-way ANOVA with Bonferroni’s post-hoc test. Probability values of P < 0.05 were considered as statistically significant (*P < 0.05, **P < 0.01, ***P < 0.001).

## Additional Information

**How to cite this article**: Pasterk, L. *et al.* Oxidized plasma albumin promotes platelet-endothelial crosstalk and endothelial tissue factor expression. *Sci. Rep.* **6**, 22104; doi: 10.1038/srep22104 (2016).

## Supplementary Material

Supplementary Information

## Figures and Tables

**Table 1:** Clinical characteristics of study subjects.

	Control (ctrl)	End stage renal disease patients	
		Hemodialysis (HD)	Kidney transplant (KTx)
**n**	14	28	29
**Age** (y)	40 (25–73)	54 (18–81)	53 (24–75)
**Male/female**	7/7	14/13	11/19
**Dialysis** (months)	–	111 ± 22	–
**Cardiovascular disease**	–	17/27	15/30
**Statins**	–	4/27	6/30
**Antihypertensive agents**	–	12/27	21/30
**Antiaggregant agents**	–	8/27	7/30
**EPO**	–	2/27	2/30
**Anticoagulants**	–	4/27	4/30
**Albumin (g/dL)**	4.7 ± 0.9	3.9 ± 0.6^a^	4.0 ± 0.8
**Hemoglobin (g/dL)**	14.3 ± 0.2	11.1 ± 2.1^a^	12.4 ± 2.2^a^
**Phosphate (mmol/L)**	1.2 ± 0.1	1.6 ± 0.3^b^	1 ± 0.2
**Creatinine (mg/dL)**	0.9 ± 0.0	8.1 ± 1.6^a^	1.6 ± 0.3^a^
**Ca**^**2+**^ **(mmol/L)**	2.4 ± 0.0	2.2 ± 0.4^b^	2.5 ± 0.5

Results are given as medians with the interquartile range or as mean ± SD Significance from the Kruskal-Wallis with Dunn post hoc test was accepted at the level of 0.001 versus control (^a^) and at the level of 0.05 versus control (^b^).
