# Supplementary material for: Oxidized plasma albumin promotes platelet-endothelial crosstalk and endothelial tissue factor expression
Source: Sci Rep. 2016 Feb 24;6:22104. doi: 10.1038/srep22104 (PMC4764952; doi:10.1038/srep22104)
Supplement: Supplementary Information [file srep22104-s1.pdf]

Supplementary Information

Oxidized plasma albumin promotes platelet-endothelial crosstalk and endothelial tissue factor expression

Lisa Pasterk, Sandra Lemesch, Bettina Leber, Markus Trieb, Sanja Curcic, Vanessa Stadlbauer, Rufina Schuligoi, Rudolf Schicho, Akos Heinemann, Gunther Marsche

Supplementary Figure

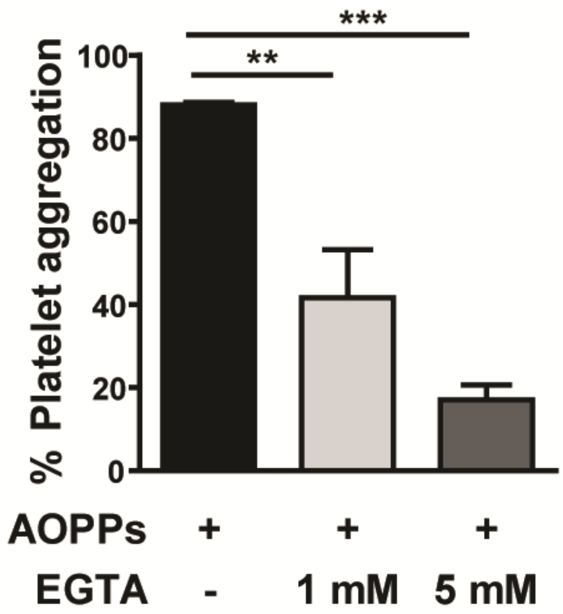

EGTA decreases AOPPs induced platelet aggregation. Platelets were preincubated with EGTA at indicated concentrations and aggregation was induced with AOPPs (100 µg/mL). All values are shown as mean + SEM. **P<0.01 and ***P<0.001 as indicated.
